# Vein networks in hydrothermal systems provide constraints for the monitoring of active volcanoes

**DOI:** 10.1038/s41598-017-00230-8

**Published:** 2017-03-10

**Authors:** Luigi Cucci, Francesca Di Luccio, Alessandra Esposito, Guido Ventura

**Affiliations:** 1 Istituto Nazionale di Geofisica e Vulcanologia, Via di Vigna Murata 605, 00143 Roma, Italy; 2 Istituto per l’Ambiente Marino Costiero, Consiglio Nazionale delle Ricerche, Calata Porta di Massa, 80133 Napoli, Italy

## Abstract

Vein networks affect the hydrothermal systems of many volcanoes, and variations in their arrangement may precede hydrothermal and volcanic eruptions. However, the long-term evolution of vein networks is often unknown because data are lacking. We analyze two gypsum-filled vein networks affecting the hydrothermal field of the active Lipari volcanic Island (Italy) to reconstruct the dynamics of the hydrothermal processes. The older network (E1) consists of sub-vertical, N-S striking veins; the younger network (E2) consists of veins without a preferred strike and dip. E2 veins have larger aperture/length, fracture density, dilatancy, and finite extension than E1. The fluid overpressure of E2 is larger than that of E1 veins, whereas the hydraulic conductance is lower. The larger number of fracture intersections in E2 slows down the fluid movement, and favors fluid interference effects and pressurization. Depths of the E1 and E2 hydrothermal sources are 0.8 km and 4.6 km, respectively. The decrease in the fluid flux, depth of the hydrothermal source, and the pressurization increase in E2 are likely associated to a magma reservoir. The decrease of fluid discharge in hydrothermal fields may reflect pressurization at depth potentially preceding hydrothermal explosions. This has significant implications for the long-term monitoring strategy of volcanoes.

## Introduction

Faults and fractures represent the pathway through which hydrothermal fluids preferentially ascend to the surface, allowing the deposition of minerals in veins and promoting self-sealing processes^1^. Vein networks may re-activate by tectonic stress and/or fluid overpressure through ‘fault-valve’ and ‘crack-seal’ mechanisms^2, 3^. Most studies on hydrothermal systems of active volcanoes are based on the geophysical and geochemical monitoring^4, 5^, but studies on the formation and evolution of vein networks are still lacking, with a few exceptions^6^. Therefore, research aimed at filling this gap may improve our understanding of the vein-controlling processes as well as of the dynamics of hydrothermal systems. This is particularly important to establish monitoring approaches and to forecast hydrothermal explosions and volcanic eruptions, which may be preceded by a sudden change in the fracture/vein networks as well as by variations in the flux of the discharged fluids. In fact, hydrothermal fields associated with active volcanoes show a large spectrum of vein networks, which range from sub-parallel to orthogonal sets and pervasively anastomosed arrangements^7, 8^. Sub-parallel arrangements form when the fluid pressure is lower than the maximum horizontal stress, whereas anastomosed networks testify a fluid pressure higher than the maximum horizontal stress^9, 10^.

**Figure 1:**
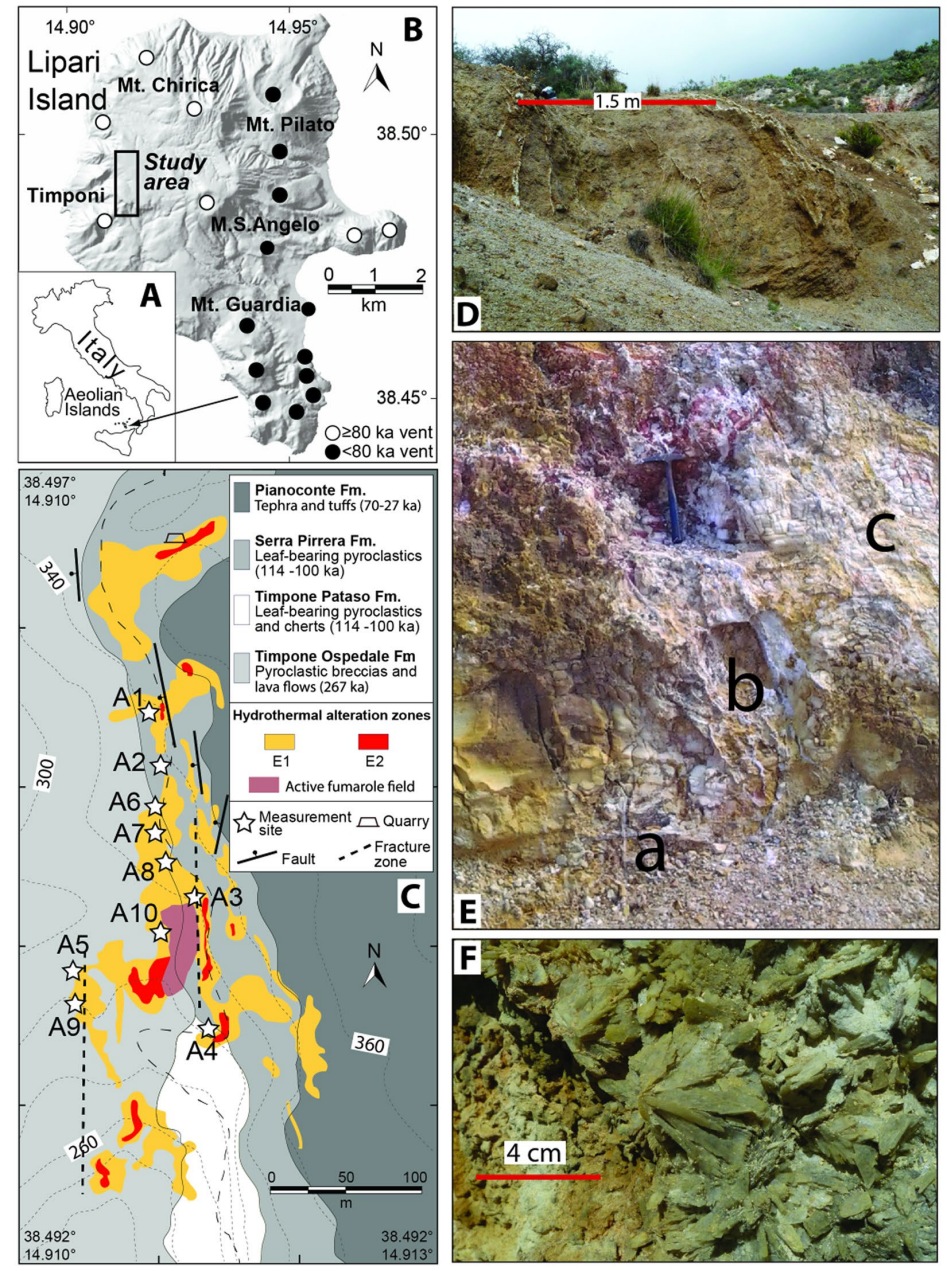
(**A**) Location of Lipari Island. (**B**) Shaded relief of Lipari Island with location of the main eruptive vents. (**C**) Geological map of the alteration zones, fractures and faults, and sites of measurements. Background colors in C indicate the different geological units along with ages^11^ as shown in the right insets. The thin dashed black line indicates the trail. (**D**) Outcrop A5 (E1 zone, view from the South) showing a system of ca. N-S striking gypsum and anhydrite filled veins with a sub-parallel arrangement affecting the welded to altered scorias of Timpone Ospedale Formation. (**E**) Outcrop A4 (E2 zone, view from the Southwest) showing cm-sized veins with orthogonal (a), quasi-anastomosed (b), and sub-parallel (c) arrangement. (**F**) Gypsum crystals filling a now inactive hydrothermal vent (measurement station A4 in C). The maps A, B, and C of this figure were generated with Adobe Illustrator CC 2015.0.0 by Adobe Systems (www.adobe.com, institution licensing).

Lipari Island (Aeolian Islands, Italy, Fig. 1A) is an ideal candidate for the study of vein networks in active volcanic and hydrothermal areas as a) it is an active volcanic complex, with eruptions occurred between 267 ka and 1220 AD^11^, b) an active hydrothermal system with associated vein network affects the western sector of the island^12^, and c) the hydrothermal alteration records a poly-phased history related to changes in the temperature and composition of the discharged fluids^13^. In this study we present original data on vein networks, which provide new insights into the processes acting in hydrothermal systems. Results have relevant implications for the interpretation of monitoring data in volcanic/hydrothermal areas.

Lipari Island consists of volcanic rocks ranging in composition from basaltic andesites to rhyolites^14^. The Lipari eruptive history can be divided in three main periods^11^: a) the early activity (267 ka to about 150 ka), mainly concentrated in the western sector and developed along N-S aligned volcanoes (Timponi in Fig. 1B); b) the 119 ka to 81 ka volcanism, developed in the central sector with the emplacements of the lavas and pyroclastics of the Monte S. Angelo and Monte Chirica volcanoes; c) the 42 ka to 1220 AD activity, which concentrates in the southern/eastern sectors and includes pyroclastics, domes and lava flows. The hydrothermal field is located in the Lipari western sector, where it forms a spatially discontinuous, N-S striking hydrothermal alteration belt (Fig. 1C). This area is interested by veins, normal faults with a prevailing NNW-SSE to N-S strike^15^, and by active fumarolic activity. Hydrothermal emissions are dominated by magmatic CO_2_ with equilibrium temperatures of 170–180 °C and SO_4_^2−^ up to 780 ppm^13, 16^. The hydrothermal alteration affects the lavas and scorias of the oldest Timponi volcanoes, the overlying pyroclastics of Monte S. Angelo, the 27 ka old Pianoconte pyroclastic deposits^11^ and the present-day soil (Fig. 1). The alteration processes developed in two main phases^13^ (a) an early one (hereafter E1, Fig. 1D), with temperature ~40 °C and pH~8 characterized by kaolinite with silica, hematite, gypsum, anhydrite (in veins), and alunite; (b) a more recent, fumarolic-type event (hereafter E2, Fig. 1E), with temperatures up to 90 °C, pH~3 and kaolinite, sulphur, gypsum with Fe-oxide globules (in veins), jarosite and sulphates (in veins and fumaroles). According to the age of the younger volcanic units affected by alteration (27 ka, Fig. 1C), we deduce that the above described alteration phases are younger than 27 ka and are still active. We cannot further constrain the age of these two phases because the age of the alteration minerals is unknown.

## Physical parameters of the vein networks

The E1 and E2 veins are characterized in the field by measuring aperture *a*, length *l*, and vein spacing *s*. Technical details and associated measurement errors are described in the section Methods. On the basis of the above mentioned measurements, we calculate, for each vein set, the percentage of extension, which is defined as ref. 9:

1 $$ext \% =\frac{{\sum }_{i=1,\ldots ,n}{a}_{i}}{L}100$$

where *a*_*i*_ indicates the aperture of the *i*-fracture and *n* is the number of fractures dissecting the profile; *L* is the length of a traverse perpendicular to the fracture strike. Due to the heterogeneity of fracture sets in natural systems, which vary in density, length and orientation, a single parameter cannot describe the physical properties of the fractured rock. The collected data, along with selected elastic parameters of the rocks, allow to estimate flow localization in fracture networks following three approaches^17^:

Fracture density (m^−1^):

2 $${F}_{den}=\frac{{\sum }_{i=1,\mathrm{...},n}a}{L}$$

where *l*_*i*_ is the length of the *i-*fracture and *A* is the area of the outcrop containing the fractures. For constant hydraulic conductivity per unit length of fracture and zero matrix permeability, equation (2) represents an indirect measure of the rock permeability.

Fracture dilatancy:

3 $${F}_{dil}=\frac{{\sum }_{i=1,\mathrm{...},n}{l}_{i}{a}_{i}}{A}$$

which is a measure of the fracture porosity or dilatancy.

Hydraulic conductance (m^4^), where the cubic law relates flow to the product of the cube of aperture (*a* ^3^) and length (*l*) of each fracture:

4 $$C=\frac{l{a}^{3}}{12}$$

where *C* expresses the ability of the rock to allow fluid flow. In the case of a flow dominated by viscous forces, *C* is related to the volumetric flux *q* (m^3^/s) through the equation^18^:

5 $$q=\frac{C}{\mu }\frac{dp}{dz}$$

where *μ* is the dynamic viscosity (kgm^−1^s^−1^) of the fluid and *dp/dz* is the pressure gradient (kgm^−2^s^−2^). Assuming an elastic rock behavior, we determine the ratio between the fluid overpressures Δ*P* in E2 (Δ*P*_*E2*_) and E1 (Δ*P*_*E1*_), which is given, in the case of a fluid-filled fracture, by ref. 18:

6 $$\frac{{\rm{\Delta }}{P}_{{E}_{2}}}{{\rm{\Delta }}{P}_{E1}}=\frac{\frac{{a}_{{E}_{2}}E}{2{l}_{{E}_{2}}(1-{\nu }^{2})}}{\frac{{a}_{{E}_{1}}E}{2{l}_{{E}_{1}}(1-{\nu }^{2})}}$$

where *E* is the Young’s modulus and *ν* is the Poisson’s ratio of the rock. Δ*P* depends on the fluid excess pressure in the source, the buoyancy effect and the differential stress in the host rock.

## Results

**Figure 2:**
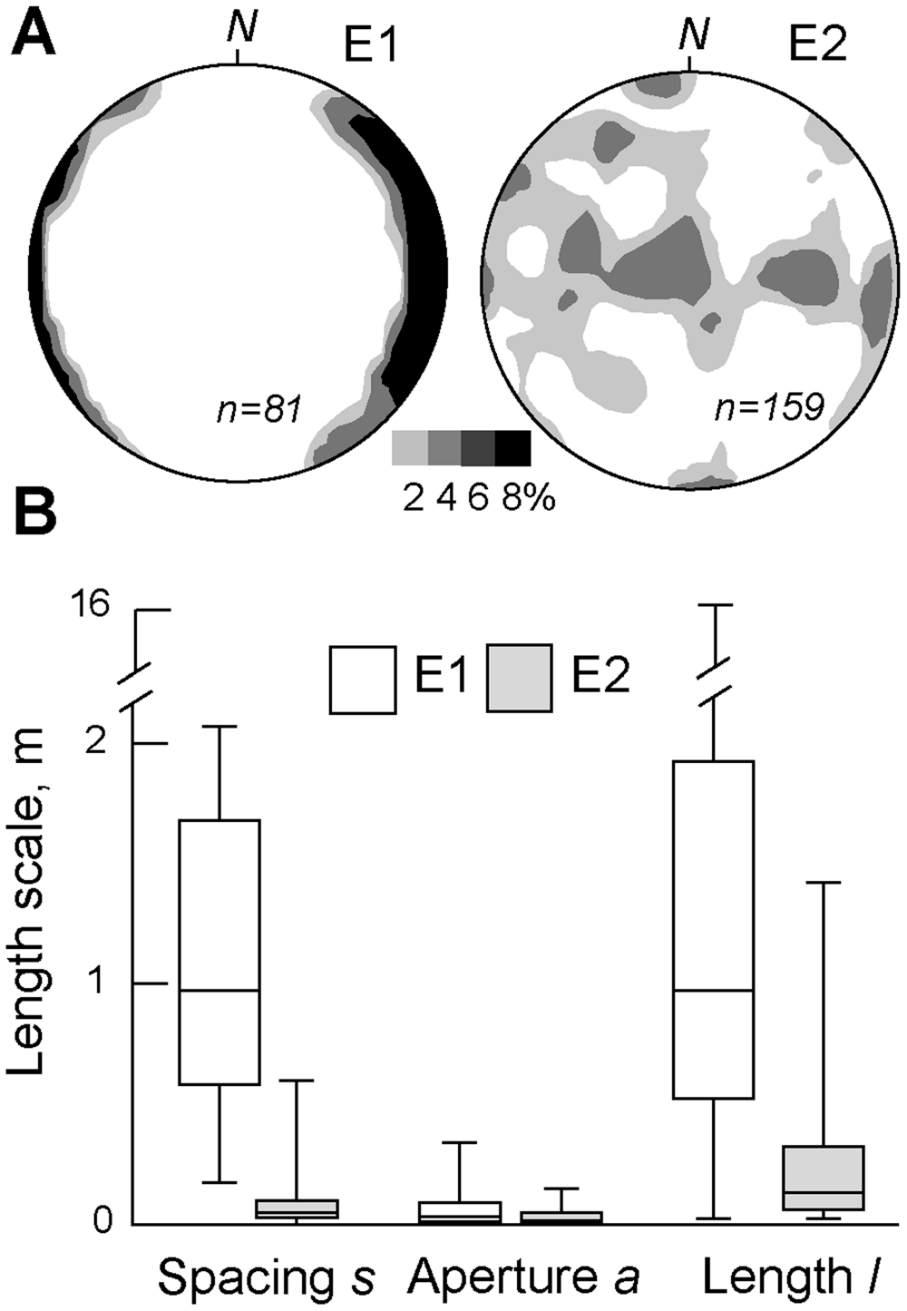
(**A**) Density plot (2–8%; equal area Schmidt net, lower hemisphere) of poles to vein walls in the E1 and E2 alteration zones. (**B**) Box plot summarizing the variations of the geometric parameters of E1 and E2 veins. Density plots were generated with GEOrient^©^ software by Rod Holcombe (http://www.holcombe.net.au/software/georient.html, academic license).

Brittle structures affecting the Lipari hydrothermal field concentrate in two main right stepping, roughly N-S striking deformation belts with an overstep of about 70 m (Fig. 1C). These structures include sub-vertical normal faults, fractures and gypsum filled veins. The easternmost belt is about 250 m long and ~50 m wide, while the western one measures 160 m in length and 15–20 m in width. The present-day fumarolic activity consists of several emission points concentrated in the overstep area (Fig. 1C). E1 alteration affects the whole hydrothermalized area, whereas E2 concentrates at the tip of the two belts and in the overstep area. Kinematic indicators of the outcropping N-S striking faults^15, 19^ indicate dip-slip movements consistent with a regional, normal stress field with sub-vertical *σ*_*1*_, sub-horizontal N-S striking *σ*_*2*_ = *σ*_*H*_, and E-W striking *σ*_*3*_ = *σ*_*h*_. The gypsum filling the E1 and E2 veins occurs in subvertical juxtaposed to sub-parallel crystals, sometimes with fibrous habit (Fig. S2B, Supplementary information). Evidences of sub-horizontal growth are lacking, suggesting a deposition during a unique major event from upward raising brine-like fluids. Frequently arranged in radial patterns, up to 3–4 cm long gypsum crystals characterize the sub-circular conduits of the fossil hydrothermal chimneys (Fig. 1F). The E1 gypsum-filled veins form sub-parallel sets, with dip ≥65° and show a prevailing N-S strike (±45°, Fig. 2A; Supplementary information). E2 veins form orthogonal sets or quasi-anastomosed networks and their orientation spans in a wide range of strike and dip values. In fact three main sets of veins may be recognized: N-S striking, sub-vertical to sub-horizontal veins, E-W striking sub-vertical veins, and NNE to NE-SW striking veins with dip of 70°–80° (Fig. 2A; Supplementary information).

Table 1 summarizes the results of the determined geometric and physical parameters extracted from the analysis of the E1 and E2 veins. Values of *s, a*, and *l* in E2 are significantly lower than those in E1 (Table 1; Fig. 2B). On the contrary, the *ext*%, *F*_*den*_ and *F*_*dil*_ values in E2 are significantly larger than those in E1; *C* values are considerably larger in E1 than in E2 (Table 1). Because of the rocks hosting the E1 and E2 veins consist of the same lithotypes, we assume that the values of *E* and *ν* do not vary between E1 and E2, and calculate Δ*P*_*E2*_/Δ*P*_*E1*_ = 4.28, being (*a/l*)_*E1*_ = 0.023 and (*a/l*)_*E1*_ = 0.1 (Table 1).

**Table 1:** Measured and calculated physical parameters of the E1 and E2 vein networks (in brackets the average value).

	E1	E2
*a* (m)	0.01–0.35(0.07)	2 10^−3^–0.15(0.03)
*l* (m)	0.1–15(2.01)	0.1–1.5(0.34)
*s* (m)	0.2–2.1(1.22)	0.6–0.01(0.1)
*ext%*	9.84–12.5	22.9–56.75
*F*_*den*_(m^−1^)	0.11	0.26
*F*_*dil*_	3.43 10^−4^	7.47 10^−4^
*C* (m^4^)	1.0 10^−2^–8.33 10^−9^	2.2 10^−6^–8.0 10^−11^

## Discussion

**Figure 3:**
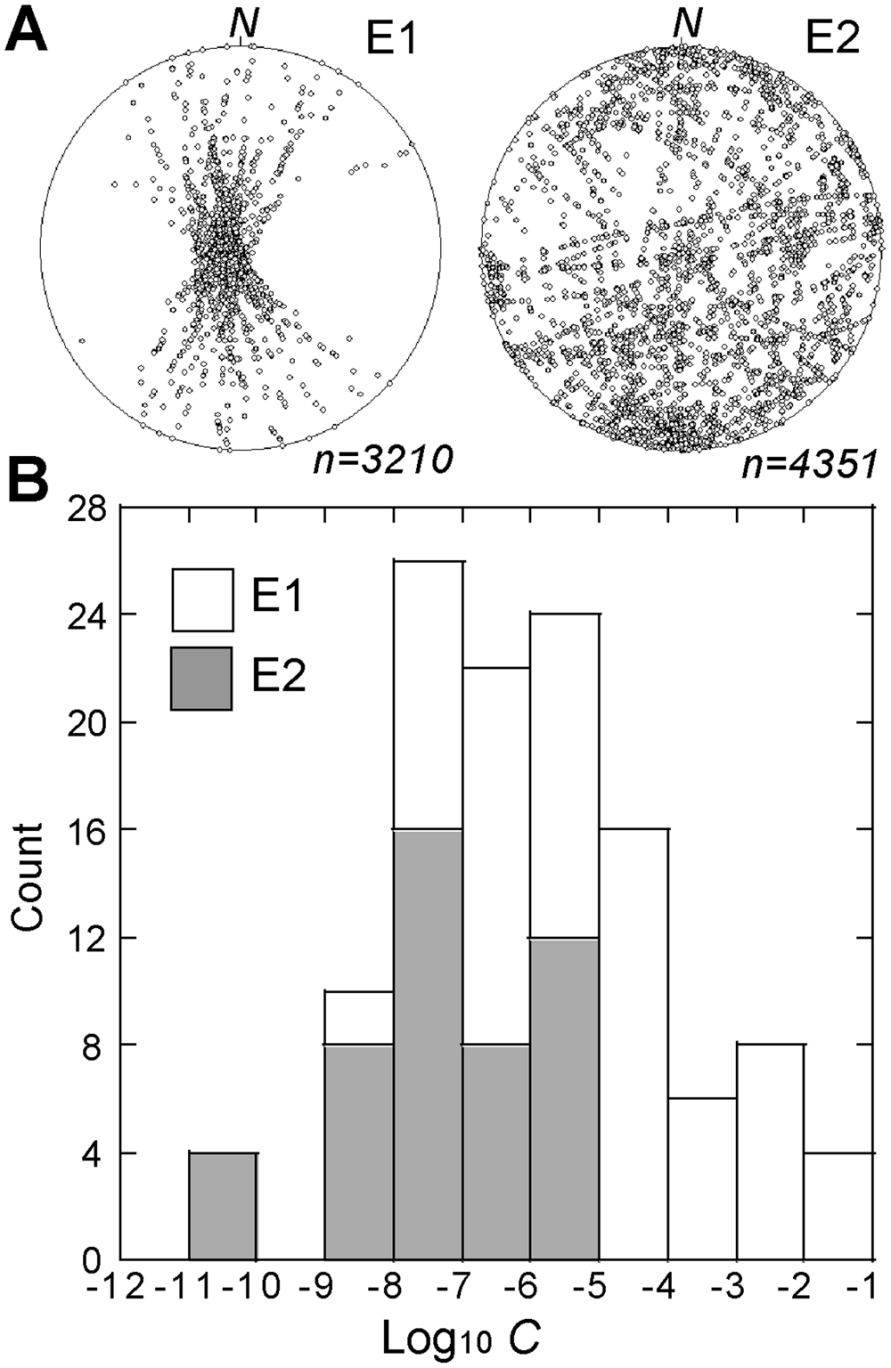
(**A**) Spatial distribution of the potential vein intersections in the E1 and E2 alteration zones (beta-plot; equal area Schmidt net, lower hemisphere). (**B**) Histogram of the calculated *C* values of the E1 and E2 veins.

The collected data indicate that the E1 and E2 vein networks differ in geometry and physical parameters (Table 1; Fig. 2). The theoretical distribution of vein intersections shows that E2 is characterized by a higher spatial dispersion and number of intersections than E1 (Fig. 3A). This indicates that a concentrated flow along a preferred N-S striking pathway characterizes E1, whereas a pervasive flow occurs in E2. The occurrence of a less concentrated flow in E2 may be due to the higher fracture density and permeability of the E2 rock mass, according to the calculated values of *F*_*den*_ and *F*_*dil*_. In addition, because in the last 27 ka the regional tectonic stress of Lipari has not changed^15, 19^, the sub-parallel arrangement of the E1 veins indicates σ_h_ = σ_3_ < Δ*P* < σ_H_ = σ_2_. In particular, the E1 veins are parallel to the local N-S striking σ_2_ and orthogonal to the E-W striking σ_3_. Therefore, the formation of these veins is controlled by the normal stress field, which is that acting at regional scale. Minor left-lateral and right-lateral shears have been also recognized in the study area, but they affect the older, 267 to 150 ka rocks^15^. However, the E1 veins form sub-parallel sets and not en-echelon or Riedel-type arrangements, as expected for right-lateral and left-lateral shear zones.

On the contrary, the lack of a preferred strike in E2, and, in particular, the concomitant occurrence of veins with an E-W strike and with sub-horizontal dip indicate Δ*P* > σ_H_ = σ_2_ and Δ*P* ≥ σ_v_ = σ_1_. Therefore, the fluid pressure is larger than the regional stress field.

The value of *C*, which is proportional to *q* and to the pressure gradient, is lower in E2 (Fig. 3B) and this could reflect a decrease in the pressure gradient. However, we exclude this hypothesis because it contrasts with the structural evidence of an increment in Δ*P* and with the determined Δ*P*_*E2*_/Δ*P*_*E1*_, which is ~4. This low value of *q* in E2 may be explained in light of experimental and numerical models of solute transport in fracture networks^20, 21^. These models show that as the number of intersections among fractures increases and the size of fractures decreases, as in E2, *q* decreases because the intersections retard the fluid passage and promote fluid interference effects^22^. Therefore, we conclude that E2 has a volumetric flux lower than E1 because of the larger number of vein intersections and smaller vein size (Figs 2B and 3A). According to equation (5), our data indicate a decrease of *q* while geochemical data^13^ record an increment in temperature (from ~40 °C in E1 to ~50°–90 °C in E2) and composition (acid and sulphur-rich fluids) due to fumarolic activity. This implies a change of the source of the hydrothermal fluids. In particular, the temperature increase and the occurrence of acid and sulphur-rich fluids could reflect the addition of a magmatic component to the hydrothermal system and a variation in the depth of the source from E1 to E2.

By assuming that inelastic zone at the tips of the Lipari veins is small relative to the linear dimensions of the vein, and in order to estimate the depth of the hydrothermal source *h*, i.e. the height of a vertical hydraulic fracture in E1 and E2, we apply the following relation^23^, which holds for fluid-filled cracks in an elastic medium:

7 $$h=\frac{a\,E}{2\,l(1-{\nu }^{2})({\rho }_{r}-{\rho }_{f})g}-\frac{{P}_{e}+{\sigma }_{d}}{({\rho }_{r}-{\rho }_{f})g}$$

where *P*_*e*_ is the excess fluid pressure at the base of the crack, *ρ*_*r*_ and *ρ*_*f*_ are the fluid and rock densities, respectively, *g* is the gravity and σ_*d*_ is the differential stress at the level where the hydrofracture is examined. Equation (7) has been previously applied to determine the depth of magma reservoirs from the geometric features of vertical magma-filled cracks (dikes)^24^, and assumes that the maximum value of (*a/l*) in a population of fluid filled cracks remains constant at depth^23^. At Lipari, we select the sub-vertical fractures (dip > 85°) with *a*_max_, which *a*lso correspond to those with the maximum (*a*/*l*), i.e. 0.023 in E1 and 0.1 in E2 (Table 1). *P*_*e*_ is assum_*e*_d to be equal to the average tensile strength of volcanic rocks^24^, i.e. 2.5 MPa, *σ*_*d*_ is the tensile stress at failure in the surface rocks, and it is fixed at 1 MPa, and *ρ*_*r*_ is assumed to be 2500 kg/m^3^. We set *ρ*_*f*_ = 1100 kg*/*m^3^ by assuming a brine-filled vein^25^. Regarding the elastic parameters *ν* and *E*, we select *ν* between 0.17 and 0.27 and *E* between 0.9 and 1.2 GPa according to the data on kaolinitized tuffs^26^. We obtain *h* = 0.88 ± 0.23 km in E1 and *h* = 4.6 ± 1.2 km in E2. As a result, the E1 hydrothermal source was located within the volcanic and sedimentary pile of Lipari, which extends between the surface and ~1.3 km depth^27^. The E2 source is deeper and stored within the crystalline basement between ~1.3 km and ~5.2 km depth^28, 29^. This conclusion is supported by geochemical data^16, 13^, which indicate the addition of a magmatic component during the E2 episode. In light of these data, the E1 alteration is related to a low *P*_*f*_, shallow hydrothermal source possibly heated by a magmatic reservoir not directly connected to it. Since Δ*P*_*E2*_/Δ*P*_*E1*_ = 4.28, the more recent E2 alteration is characterized by significant pressurization of a deeper, hydrothermal system (*h* = 4.6 ± 1.2 km) possibly connected to a magma reservoir.

The emplacement of sub-horizontal veins in E2 implies that an uplift due to the formation of sub-horizontal veins has occurred during the E2 hydrofracturing event. Based on the outcropping veins, we estimate this cumulative vertical opening by summing the apertures of the sub-horizontal veins. By selecting 27 vertically juxtaposed veins with dip ≤25°, we obtain a minimum value of ca. 0.62 m in E2.

As concerns the time scale for the sealing of the Lipari veins by gypsum/anhydrite filling, the available estimates of growth rate of these minerals along a fracture span from 0.03 to 315 mm/yr^30^, with an average value of 3 mm/yr. This large variation reflects the complexity of the growth process, which depends on the saturation of the solution, pH, temperature, fluid pressure, and applied stress. The average value of 3 mm/yr is of the same order of magnitude of the 1.3 mm/yr hydrothermal alteration rate estimated at Lipari^13^. Therefore, to estimate the time scale required for the sealing of the Lipari veins, we divide the aperture values of the E1 and E2 veins (Table 1) by the growth rate of 3 mm/yr^30^. The obtained results are: 3 to 117 yr for the E1 veins (average 23 yr) and 0.7 to 50 yr for E2 (average 10 yr). These values, although affected by large uncertainties, suggest that the time scale for the sealing of the Lipari hydrothermal veins is of years and the time span for the sealing of the E1 veins is larger than that required for E2. As known, sealing processes reduce the rock permeability, increase the pressure of the hydrothermal fluids at depth, and may favor uplift episodes^31^. Accordingly, time scales for the sealing of the Lipari veins, and, possibly, for the local uplift, are comparable to those estimated for the uplift episodes observed at other active volcanoes, e.g. in 1982–1984 and 1400–1538 at Campi Flegrei (Italy)^32^, and in 1923–1985 at Yellowstone Caldera^5^.

The present-day Lipari gas emissions concentrate in the overstep zone between the two main N-S striking deformation belts affecting the hydrothermal area (Fig. 1). These overstep zones represent dynamically maintained interaction areas where active fracturing, re-opening of fluid pathway, and fluid discharge likely occur, even though self-sealing processes operate^28, 29^.

Our analysis of the Lipari vein networks has implications for the monitoring strategies of hydrothermal systems and to better understand the deformation processes related to fluid upraising and pressurization. The pressurization of the Lipari hydrothermal system produced an unrest episode possibly associated with the emplacement of a passively degassing shallow magma reservoir. Similar unrests, including uplifts, are recorded, among others, at the active Campi Flegrei and Yellowstone calderas^4, 33^. At Lipari, we provide for the first time the field-based evidence that the hydraulic conductance and thus the volumetric flux in a hydrothermal field decrease with the increase of the number of intersections of fluid-filled fractures. This may be caused by crack growth processes and/or formation of small cracks (low *l* and *a*) acting as bridges among the older and larger ones. An increase in the number of fracture intersections favors longer residence time of brine-like fluids, so promoting deposition and self-sealing processes^34^ and an overall increase of the fluid pressure at depth. As a result, a decrease in the fluid discharge in active hydrothermal fields does not unequivocally indicate a depressurization event, but, on the contrary, could be a sign of pressurization at depth. In addition, as recorded at Lipari, the formation of horizontal veins may induce an uplift.

Our conclusions support the results from physical models of uplift associated to hydrothermal systems^35^ and allow to explain the data collected before the 2006 phreatic eruptions at Poas volcano (Costa Rica)^36^ and at Campi Flegrei between 1983 and 2003^37^. In detail, physical models^35^ show that a low discharge of hydrothermal fluids associated to uplift reflects high pore pressures at depth related to reduction in permeability by self-sealing processes. At Poas volcano, phreatic eruptions occurred in 2006 and were not anticipated by significant changes in the concentration of sulfates in the crater lake between 1995 and 2005^36^, thus suggesting a pressurization of the hydrothermal system at depth before the eruption. At Campi Flegrei, the increase of CO_2_/H_2_O occurred during the four unrest episodes between 1983 and 2003 is delayed of hundred of days with respect to the beginning of the uplift and seismic phases^37^. This delay indicates that the early injection of CO_2_ at depth pressurizes a sealed hydrothermal system and induces an uplift^36^; the later release of CO_2_ to the surface occurs when the system increases its permeability because of the opening of fractures due to earthquakes. As a conclusion, low discharge of hydrothermal fluids may reflect pressurization processes and precede hydrothermal explosions like those occurred at Nisyros Island, Greece in 1871–1888, at Porkchop Geyser in Norris Geyser Basin, USA in 1989, and at Poás volcano, Costa Rica in 2006. Phreatic and hydrothermal explosions may thus interrupt cycles of self-sealing and vein network formation. The recurrence time of such cycles and of the unrest episodes may help for an evaluation of the hazard associated to hydrothermal/phreatic explosions and gas discharge. As a general conclusion, the long term monitoring of vein networks in volcanic areas can give useful information on the dynamics of hydrothermal systems and allow us to constrain the interpretation of the geochemical and geophysical signals.

## Methods

We conducted a field mapping of the two alteration zones and structural measurements of all the outcropping veins in the Lipari hydrothermal area. Measurements are taken in ten main sites, which include the E1 or E2 alterations (Fig. 1). We measured the fracture spacing *s* (m), the maximum vein aperture (thickness) *a* (m) and length *l* (m). In many cases, the vein thickness is constant along *l* and decreases at the vein tips. *s* is measured on profiles oriented perpendicularly to the local preferred strike of the vein network. Profiles of different orientation are selected in the case of veins with orthogonal strikes. *a* is measured on sections orthogonal to the vein walls. The measurement error is ±2° on vein strike and dip. The measurements of lengths (e.g., thickness and length of veins) are ±1 mm for a length scale < 40 cm and ±2 mm for a scale > 40 cm and <180 cm; for a length scale > 180 cm, the error is ±4 mm. The parameters of the statistical distribution of the E1 veins are: Mean Principal Orientation = 84/269; Mean Resultant direction = 82–261; Mean Resultant length = 0.74 (Variance = 0.26). Shape parameter, gamma = 0.35; Strength parameter, zeta = 5.00; Eigenvalue log ratios: e_2_/e_1_ = 3.70|e_3_/e_2_ = 1.30.The parameters of the statistical distribution of the E2 veins are: Mean Principal Orientation = 40/118; Mean Resultant direction = 17–157; Mean Resultant length = 0.56 (Variance = 0.44); Shape parameter, gamma = 0.52; Strength parameter, zeta = 0.74 Eigenvalue log ratios: e_2_/e_1_ = 0.49| e_3_/e_2_ = 0.25.

## Electronic supplementary material

Supplementary Info
