# Supplementary material for: Vein networks in hydrothermal systems provide constraints for the monitoring of active volcanoes
Source: Sci Rep. 2017 Mar 10;7:146. doi: 10.1038/s41598-017-00230-8 (PMC5428009; doi:10.1038/s41598-017-00230-8)
Supplement: Supplementary file 1 — Supplementary Info [file 41598_2017_230_MOESM1_ESM.pdf]

Vein networks in hydrothermal systems provide constraints for the monitoring of active volcanoes

Luigi Cucci[1], Francesca Di Luccio[1], Alessandra Esposito[1], Guido Ventura[1,2]

[1]Istituto Nazionale di Geofisica e Vulcanologia, Via di Vigna Murata 605, 00143, Roma, Italy
[2]Istituto per l'Ambiente Marino Costiero, Consiglio Nazionale delle Ricerche, Calata Porta di Massa, 80133 Napoli, Italy

Supplementary information

Fig. 1S. Poles to vein walls in the different measurement stations of Fig. 1.

Fig. 2S. Photos representative of the E1 and E2 alteration zones, veins, and crystals.

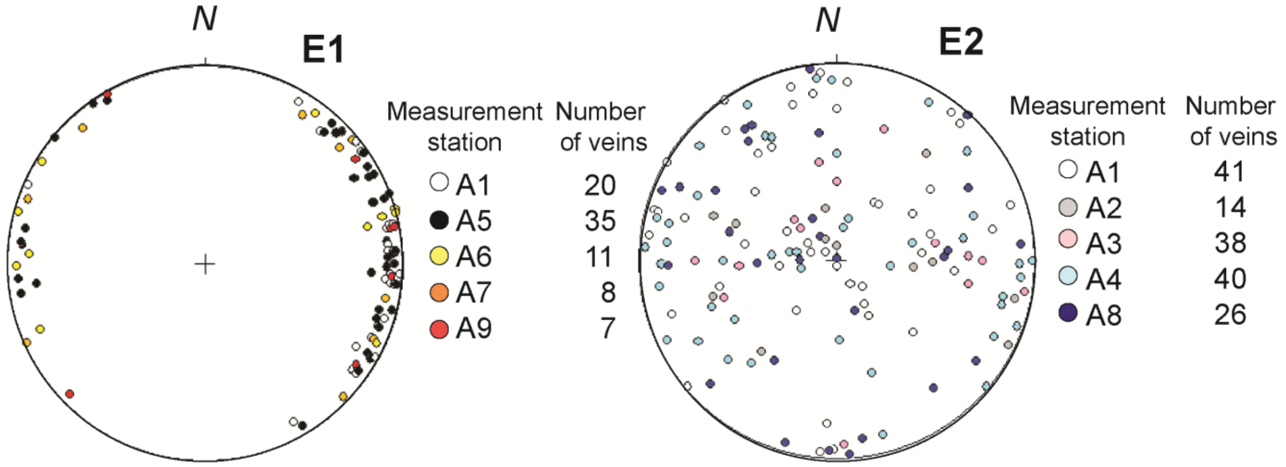

Figure 1S. Poles to vein walls in the different measurement stations of Fig. 1 of the main text and number of veins in each outcrop. E1 and E2 are the alteration zones described in the text.

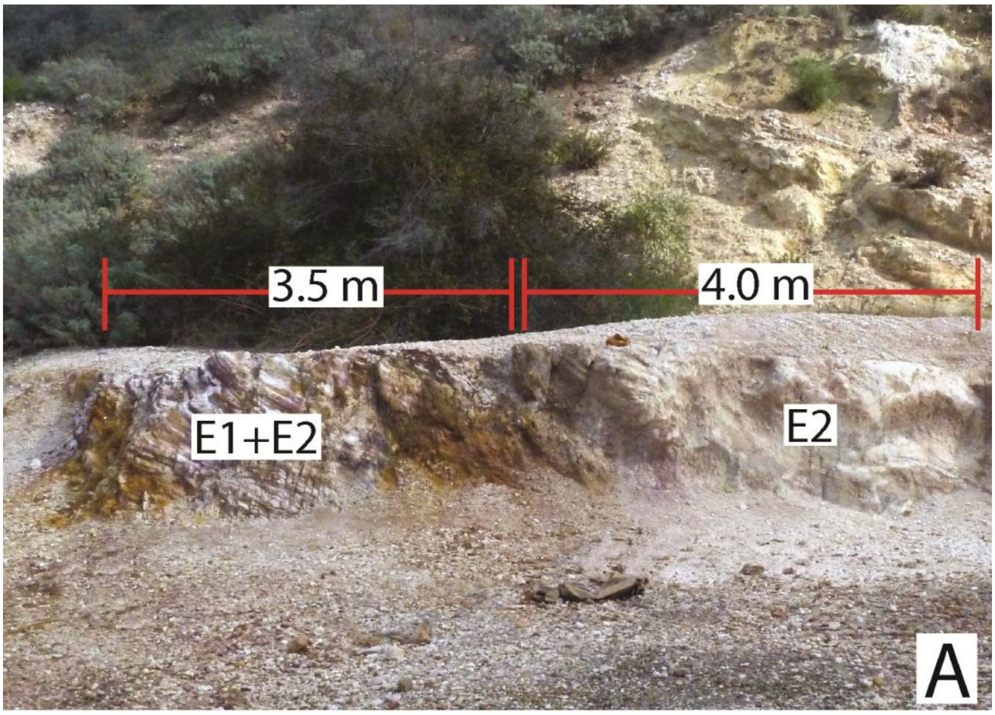

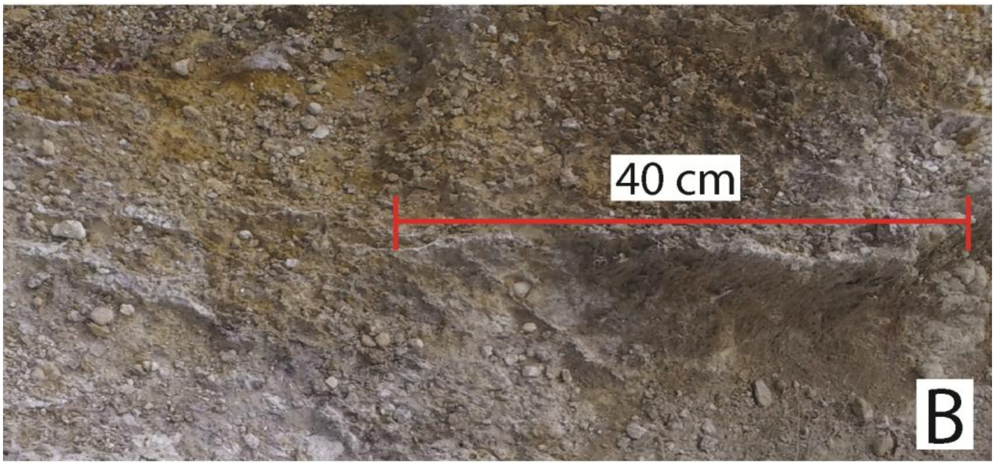

Figure 2S. A. Outcrop A1 (view from the South; Fig. 1 of the text for location) showing the E1 (kaolin, white color) and E2 (sulphates, yellow color) alteration zone. The E2 zone mainly develops within a fault damage zone and intrudes the E1 zone. The alteration affects the pyroclastics of the Serra Pirrera Formation (strike N45°E, Dip 30°SE). The whitish right portion is pervasively altered and shows highly fractured sand with brecciated clasts less than 1 cm wide. B. View from above of E1 gypsum crystals with a sub-vertical fibrous habit filling a ~ N-S striking vein in station A5.
